# The Danger Lurking in the Bill

**Published:** 1919-04-19

**Authors:** 


### THE MATRONS' AND SISTERS' DEPARTMENT.

### THE DANGER LURKING IN THE BILL.

It is evident that the Government is taking a keen and practical interest in the Bill for the Registration of Nurses. The main battles in Committee are being fought, as was anticipated, over the composition of the General Nursing Council and its precursor the Provisional Council. It is hardly possible to exaggerate the importance of due representation on these bodies. Unless the balance be preserved there is grave danger that extremists may wrest the power into their own hands, develop the future of nursing on impracticable lines, and render Registration abortive by making it possible for only a mere fraction of nurses. To attempt to force up the standard of examination above the general level of education in the country would be to compel many, perhaps even the majority of nurse training-schools to stand outside the measure. It would result in the very course which the advocates of Registration have always deprecated—the introduction of a double standard. On the one hand we should have an exclusive body of registered nurses eligible for teaching and other good posts, securing, in fact, all the plums of the profession, able to charge very high fees and monopolising the best medical and surgical practice. On the other hand we should have a very large body of women, more or less well trained in a practical manner, but quite unsupervised. It would be represented to the public by doctors and others that these were fully as good as the registered brand of nurses, and the public, which excessively dislikes any monopoly making for high charges, would readily believe this to be true.

The success of Registration depends, as we have constantly pointed out, on its proving acceptable not merely to a comparatively small group of highly trained women, but to the main body of the profession. It must not soar above the heads of the rank and file. It must be a strictly practical measure comprising provisions which it is possible to carry out in the interests of the entire body of women who adopt nursing as their career. It is nurses who are themselves skilled in the difficult art of attracting, retaining, and training probationers who are best qualified to see all round this subject and avoid the fatal mistake of erecting too formidable a barrier before the profession.

The war has brought before the world the national importance of the nurses. A nursing service must be kept up at a prodigious size to be enabled to furnish, as ours has done, 20,000 women for military and naval duties. It will not do to restrict it. Those who have thought most deeply on the subject have reached the conclusion that a standard of education can be set up which will enable every training-school of due size at present turning out nurses to prepare its pupils successfully for Registration; that an examination can be devised which shall test nursing abilities without putting too great a strain on educational attainments. It is an exceedingly nice matter. On the success of the Council in fixing the standard, so that it shall be a real standard yet not too exclusive a one, depends the success of Registration.

It will be seen how vital, then, is the matter of the Council. If it came to be constituted in such a manner that those divorced from present responsibilities, mere theorists with a fixed idea of making the nursing service a strict preserve for one type of woman, had the preponderance, the measure might come in the course of a few years to justify the gloomy prognostics in which Ierne indulged in our last issue.

While holding to the conviction that the standard of examination set up by the Nursing Council ought not to be higher than can be attained by women educated under the present admittedly low educational standard, we look forward to its gradual and very decided rise as national methods of education improve. We look forward to the creation also of higher special grades of nurses, fitted for teaching and superintendence. Once secure a sound workable system which shall guarantee to the public that the average nurse knows her business and has passed creditably through suitable tests—once let this be achieved—and higher examinations can be instituted for those who desire to qualify for higher posts.

The great danger of non-compulsory registration is lest it should become, as in many of the States of America, a mere counsel of perfection for the few instead of a measure giving effectual protection to nurses in general and to the public.

